# A Fault Diagnosis Method for Transmission Networks Based on Multi-Source Information Fusion

**DOI:** 10.3390/e28060709

**Published:** 2026-06-20

**Authors:** Shifu Gu, Xiaotian Chen, Tao Wang, Quanlin Leng, Chunyu Zhou

**Affiliations:** 1 School of Electrical Engineering and Electronic Information, Xihua University, Chengdu 610039, China; 2 Neijiang Power Supply Company, State Grid Sichuan Electric Power Company, Neijiang 641199, China

**Keywords:** multi-source, fault diagnosis, meteorological factors, fault degree

## Abstract

In order to solve the miscalculation problem caused by the distortion and loss of fault information caused by the traditional transmission grid fault diagnosis method due to the severe meteorological environment, a transmission grid fault diagnosis method based on multi-source information fusion is proposed. Firstly, the pulse fault degree, amplitude fault degree and meteorological fault degree are obtained by analyzing the switching, electrical and meteorological information from multiple sources using the binary reasoning spiking neural P systems, Hilbert–Huang transform and meteorological fusion methods, respectively. Then, the fault diagnosis results are obtained by fusing the various fault degrees using the analytic hierarchy process. Finally, simulation experiments are conducted on the standard IEEE39-bus system built by PSCAD simulation software, and the results verify the feasibility and effectiveness of the proposed diagnosis method in this paper.

## 1. Introduction

Power system fault diagnosis is an important foundation for achieving rapid recovery after a smart grid fault. Based on the fault information received by the control center, it determines the cause of line faults and fault locations through diagnostic methods, providing a decision basis for grid maintenance operators to deal with faults in a timely and accurate manner and quickly restore power supply [1,2].

Therefore, researchers have gradually proposed grid fault diagnosis methods for decision aids, such as expert systems [3], analytic models [4,5], artificial neural networks [6,7], Bayesian networks [8,9,10], Petri nets [11], and spiking neural P systems [12]. These methods mainly focus on fault diagnosis by using information from the actions of transmission grid protection devices (i.e., protective relays and circuit breakers). Although they improve the accuracy of grid fault diagnosis by using different techniques, they are still not effective in eliminating the negative impact caused by the misoperation and rejection of protection devices. And this negative impact will probably lead to misdiagnosis by the diagnostic methods. Especially in the severe meteorological environment, the possibility of the protection device misoperation and rejection is greater. Therefore, the single switching fault diagnosis method has certain limitations.

In transmission network fault diagnosis, fault-related information is mainly derived from three data sources, namely switching quantity, electrical quantity and meteorological data. Switching quantity refers to the status information generated by protection devices and circuit breakers in the power system, typically represented as discrete signals such as 0/1 or operated/not operated. This type of data is the main basis for traditional fault diagnosis methods, but its reliability is easily affected by device rejection, misoperation, and information loss or distortion during transmission. Electrical quantity refers to continuous waveform data, such as voltage and current, on transmission lines or buses before and after a fault occurs. Electrical quantities change faster than switching quantities and are not affected by the operation logic of protection devices, offering higher reliability and fault characterization capability. Meteorological data refers to real-time weather information of the transmission line environment, including lightning, rainfall, wind speed, etc. Severe meteorological conditions are important factors leading to line faults and abnormal protection device operation. Introducing meteorological data helps improve the robustness of the diagnostic method.

When a fault occurs in the transmission network, the electrical quantity changes and the protection device acts immediately afterwards. Electrical quantities are closer to the occurrence of faults than switching quantities, and electrical quantity information does not have the misoperation and rejection of the device, so it has higher reliability and fault tolerance. Therefore, the use of electrical quantity information can effectively eliminate the negative effects of the protection device rejection and misoperation [13]. In order to effectively combine switching quantities and electrical quantities, Ref. [14] first diagnosed switching quantities using an optimized model improved by the coverage set theory, then used wavelet methods to extract the fault degree of electrical quantities of fault recordings, and finally obtained the diagnosis results based on the improved evidence theory. The Ref. [15] used fuzzy Petri nets and Hilbert–Huang transform to extract the fault degree of switching quantities and electrical quantities, respectively, and then fused the results based on evidence theory to make fault state judgments. Compared with the fault diagnosis method using only switching quantities, the diagnosis method of multi-source information fusion improves the accuracy and fault tolerance of fault diagnosis [16]. However, the above multi-source information fusion method requires modeling of the whole system and electrical quantity information of all lines, which increases the modeling complexity and reduces the fault diagnosis efficiency as well as the practicality of the diagnosis method.

In a severe meteorological environment, power system protection devices are more prone to misoperation and refusal to operate; and information collection equipment may be damaged, resulting in the loss of fault information. In addition, the severe meteorological environment also tends to reduce the reliability of power communication equipment, thus increasing the possibility of fault information distortion. The misoperation and rejection of protection devices, as well as the loss and distortion of information will lead to increased uncertainty in the fault information used for diagnosis, thus affecting the accuracy of the diagnosis method. However, existing research work has less consideration of the above effects of meteorological environment on fault information.

Therefore, this paper proposes a transmission grid fault diagnosis method based on multi-source information fusion. The main contributions of this paper are described as follows.

(1) To address the issue that traditional single-source fault diagnosis methods are vulnerable to protection device misoperation, operation refusal, and information uncertainty, this paper proposes a multi-source information fusion-based fault diagnosis method for transmission networks. The proposed method integrates three heterogeneous information sources, namely switching quantities, electrical quantities, and meteorological data. Correspondingly, three fault degrees are extracted using the binary reasoning spiking neural P system, the Hilbert–Huang transform combined with an improved Sigmoid criterion, and a meteorological fault assessment model, respectively. On this basis, the analytic hierarchy process (AHP) is employed for decision-level fusion to achieve optimal weighting of fault information from different sources. Experimental results demonstrate that the proposed method exhibits strong fault tolerance under conditions of missing or erroneous information. Moreover, by adopting an independent single-line modeling strategy rather than global network modeling, the method effectively reduces modeling complexity while significantly improving diagnostic accuracy and engineering practicality.

(2) To overcome the lack of quantitative evaluation capability for severe weather impacts in existing fault diagnosis methods, this paper proposes a comprehensive meteorological fault assessment method incorporating weather-related fault rates. Specifically, a fault-rate database is constructed for key meteorological factors, including lightning, rainfall, and wind. Based on real-time meteorological conditions, the corresponding fault rate for each factor is determined, and a probabilistic product model is subsequently employed to calculate the overall meteorological fault degree of the transmission line. By transforming adverse weather effects into quantifiable diagnostic indicators, the proposed method overcomes the limitation of traditional approaches that rely solely on switching states and electrical quantities. Experimental results demonstrate that incorporating meteorological fault degree as an independent information source within the AHP-based fusion framework significantly enhances the robustness and fault tolerance of the diagnosis model under severe weather conditions.

(3) To mitigate the high modeling complexity caused by the dependence on full-system electrical measurements in traditional multi-source diagnosis methods, this paper proposes a localized electrical fault degree criterion based on an improved Sigmoid function. Unlike conventional approaches that require electrical information from the entire transmission network, the proposed criterion only utilizes the voltage information of the target transmission line itself. While maintaining satisfactory diagnostic performance, the proposed approach significantly reduces both information acquisition requirements and computational burden, thereby greatly improving the deployment efficiency and practical applicability of the diagnosis method in engineering applications.

The remainder of this paper is organized as follows: Section 2 gives the basic theories involved in the multi-source information fusion method. In Section 3, the transmission grid fault diagnosis method with multi-source information fusion is presented. After that, the proposed method is applied to the standard IEEE39-bus system built by PSCAD software in Section 4 to illustrate its feasibility and effectiveness. Finally, conclusions are drawn in Section 5.

## 2. Basic Theory

### 2.1. Binary Reasoning Spiking Neural P Systems

**Definition** **1.** *A binary reasoning spiking neural P systems (brSNPS) [12] with a degree of $m\left(m\geq 1\right)$ is a tuple*

$$\Pi =\left(O,\sigma_{1},...,\sigma_{m},syn,in,out\right)$$

*where*

*(1) O={a} is a set of singleton alphabets, and a denotes a pulse.*

*(2) $\sigma_{i}=\left(\theta_{i},r_{i}\right)$, 1≤i≤d, denotes the i-th propositional neuron; $\sigma_{j}=\left(\theta_{j},r_{j}\right)$, 1≤j≤k, denotes the j-th rule neuron, and d+k=m, where*

*(a) $\theta_{i}$(or $\theta_{j}$) is a natural number with a value of 0 or 1, denotes its corresponding internal neuronal pulse value.*

*(b) $r_{i}$ denotes the firing rule of the propositional neuron $\sigma_{i}$, which is in the form of $E/a^{\theta }\to a^{\theta }$, where θ is a natural number of 0 or 1; The fire rule can be applied if and only if it receives at least one spike. When the firing rule is executed, the propositional neuron $\sigma_{i}$ will consume a spike $a^{\theta }$ with a pulse value θ and simultaneously generate a new spike $a^{\theta }$ with the same pulse value θ, and emit $a^{\theta }$ to the postsynaptic neuron of $\sigma_{i}$. If $\sigma_{i}$ has no postsynaptic neuron, it will only accumulate pulse values.*

*(c) $r_{j}$ denotes the firing rule of the rule neuron $\sigma_{j}$, which is in the form of $E/a^{\left(\theta_{1},...,\theta_{s}\right)}\to a^{\beta }\left(s\geq 1\right)$, where both θ and β are natural numbers of 0 or 1; The fire rule can be applied if and only if it receives at least s spikes. When the firing rule is executed, the rule neuron $\sigma_{j}$ will consume s spikes while generating a new spike $a^{\beta }$ with the pulse value β, and emit $a^{\beta }$ to the postsynaptic neuron of $\sigma_{j}$. For propositional neurons, the internal parameter σ represents the current pulse value held by the neuron, which is either 0 or 1. It indicates the truth status of a certain proposition. For rule neurons, the internal parameter σ also represents a pulse value, but it denotes the output pulse value generated after the rule neuron executes its firing rule. This output is then transmitted to downstream propositional neurons as an inference result. The essential difference lies in the fact that σ in a propositional neuron reflects its state, whereas σ in a rule neuron reflects the result of its firing behavior. In the computational process, propositional neurons typically represent facts or conclusions, while rule neurons implement logical reasoning.*

*(3) syn⊆{1,...,m}×{1,...,m} denotes the directed synaptic connectivity relationship between neurons in *Π*, where i≠j for all (i,j)∈syn,1≤i,j≤m.*

*(4) in,out⊆{1,...,m} denote the sets of input and output neurons, respectively.*

*A brSNPS system contains one kind of proposition neurons and three types of rule neurons. Their definition and calculation of pulse values θ (0 or 1) vary depending on the neuron type. Detailed characteristics and computation rules are described in Refs. [17,18].*

### 2.2. Hilbert–Huang Transform

The Hilbert–Huang Transform (HHT) [19,20] is a mathematical tool that can efficiently handle non-smooth data signals, and its analysis process is fully adaptive and suitable for analyzing telemetry information during power system faults. Two main steps of HHT [21,22]:

(1) Empirical Mode Decomposition (EMD): the original signal is empirically decomposed to obtain multiple Intrinsic Mode Function (IMF) components. EMD is a decomposition of a non-smooth data signal into several different smooth data, where a set of smooth data signals are the first-order IMF components.

(2) Hilbert Spectral Analysis (HAS): Hilbert transform (HT) is performed on each order of IMF components to obtain the instantaneous amplitude and instantaneous frequency of each order of IMF components. Specifically, the Hilbert transform is defined as Equation (1).

(1) $$f\left(t\right)=\frac{1}{\pi }\int_{-\infty }^{\infty }\frac{x(\tau )}{t-\tau }d\tau$$

where x(t) denotes the original function signal. Then the analytic signal F(t) of the original function signal is as shown in Equation (2).

(2) $$F\left(t\right)=x\left(t\right)+jf\left(t\right)=A\left(t\right)e^{j\omega (t)}$$

where A(t) denotes the instantaneous amplitude of the signal, i.e., $A\left(t\right)\;=\;\sqrt{x^{2}\left(t\right)+y^{2}\left(t\right)}$; ω(t) denotes the instantaneous frequency, i.e., $\omega \left(t\right)\;=\;\arctan\frac{f(t)}{x(t)}$.

### 2.3. Analytic Hierarchy Process

Analytic hierarchy process (AHP) [23,24] refers to the decomposition of a complex objective into multiple sub-objectives to singularize and multiply complex objectives. The essence of this method is to consider multiple factors affecting the total objective, and then decompose the same level of factors as sub-objectives at the same level and layer by layer, and finally convert them into simple single objectives or easily solvable multiple objectives. The solution process of the analytic hierarchy process is as follows:

(1) Build hierarchical model

The total objective layer is used as the first layer, i.e., the objective of the solution. The second layer is the factors affecting the objective, as the first layer, and the individual factors in the second layer are used as the objective. Then, the decomposition is done in successive layers until the final layer of known factors.

(2) Construction of judgment matrix

According to the relative importance of every two factors at the same level, given the corresponding ordinal number, the relationship between the relative importance of every two factors is shown in Table 1 [25]. Judgment matrix $V={\left[v_{ij}\right]}_{u\times u}$ was constructed from the comparison results between the factors.

(3) Calculate the weight vector of factors

The judgment matrix *V* is calculated according to Equation (3) to obtain the weight vector $W={\left[W_{i}\right]}_{u\times 1}$ of factors, which

(3) $$W_{i}=\frac{\sqrt[{u}]{\prod_{j=1}^{u}v_{ij}}}{\sum_{i=1}^{u}\sqrt[{u}]{\prod_{j=1}^{u}v_{ij}}}$$

where $W_{i}$ denotes the weight of i-th factors; $v_{ij}$ denotes the importance of i-th factors relative to j-th factors; *u* denotes the number of factors involved. In both the numerator and denominator, *u* denotes the *u*-th root.

## 3. Transmission Grid Fault Diagnosis with Multi-Source Information Fusion

**Figure 1:**
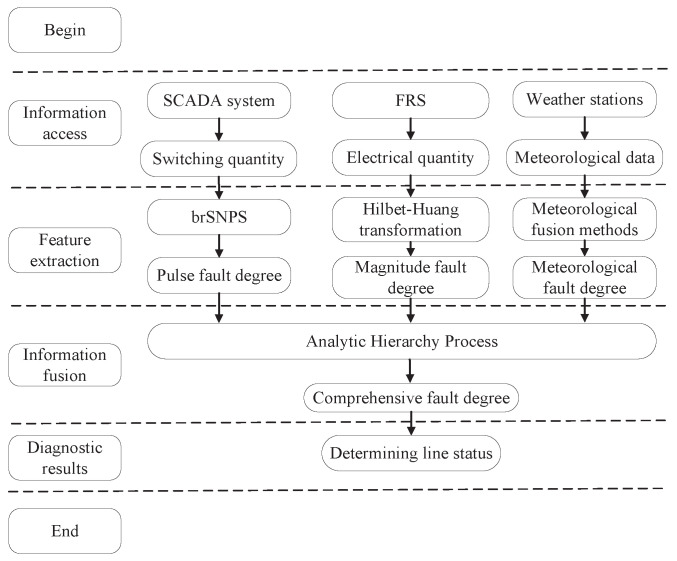
Flowchart of proposed method.

This section proposes a fault diagnosis method with multi-source information fusion, whose flowchart is shown in Figure 1.

Detailed steps of the proposed method are described as follows:

Step (1): Acquisition of fault diagnosis information. Obtain the switching information, electrical information and meteorological data of the location of the transmission network line at the time of fault from the SCADA system, fault recording system and meteorological station, respectively; and determine the fault area and identify the suspected fault line using the junction analysis method.

Step (2): Feature extraction of the acquired diagnostic information. The switching, electrical and meteorological data acquired in step (1) are subjected to feature extraction, and the pulse fault degree, amplitude fault degree and meteorological fault degree of the suspected faulty line are calculated respectively. Amplitude fault degree is a continuous value calculated by analyzing voltage waveform data using the HHT and improved Sigmoid function, quantifying the severity of a fault on a line or bus. A value closer to 1 indicates a higher probability of fault. Meteorological fault degree is a comprehensive value calculated using meteorological data and the failure rates corresponding to different meteorological levels via the formula, representing the risk level of a line fault caused by meteorological conditions.

(a) Pulse fault degree

Based on the information feature extraction of the acquired switching quantities by brSNPS, the switching fault degree corresponding to the suspected faulty line, i.e., the pulse fault degree, is obtained. Switching fault degree is the pulse value output after reasoning on switching information using the brSNPS model, representing the preliminary judgment of whether a fault exists on a given line or bus.

(b) Amplitude fault degree

Firstly, the electrical quantity data obtained are processed according to the HHT method. Then, the electrical quantity fault degree corresponding to the suspected faulty line is obtained based on the improved Sigmoid function, i.e., the amplitude fault degree. The specific steps are described as follows:

(i) First, the original voltage signal of the suspected fault line is empirically modal decomposed to obtain the multi-order IMF components of the original voltage signal. Then, it is Hilbert transformed to obtain the instantaneous amplitude of each order IMF component. Finally, the total amplitude of the IMF components before and after the fault is calculated according to Equations (4) and (5), respectively.

(4) $$A_{l}^{f}=\frac{\sqrt{\sum_{\rho =1}^{n}{\left(\frac{\sum_{P=P_{J}}^{P_{f}}\frac{A_{l\rho }\left(P\right)}{\sqrt{2}}}{N_{f}}\right)}^{2}}}{n}$$

(5) $$A_{l}^{g}=\frac{\sqrt{\sum_{\rho =1}^{n}{\left(\frac{\sum_{P=P_{f}}^{P_{g}}\frac{A_{l\rho }\left(P\right)}{\sqrt{2}}}{N_{g}}\right)}^{2}}}{n}$$

where $A_{l}^{f}$ and $A_{l}^{g}$ denote the total amplitude of the IMF component before and after the fault of the line *l*, respectively; $P_{J}$, $P_{f}$ and $P_{g}$ denote the beginning of the sampling point of the first three cycles of the waveform when the line fault occurs, the sampling point when the fault occurs and the last sampling point of the waveform after the three cycles of the fault, respectively; $A_{l\rho }$ denotes the amplitude of the IMF component of the ρ(1≤ρ≤n) order of the line *l*; *n* denotes the order of the IMF component; $N_{f}$ and $N_{g}$ denote the number of sampling points within the three cycles of the waveform before and after the line fault, respectively.

(ii) The relative voltage magnitude change before and after the line l fault is calculated according to Equation (6), i.e., $\eta_{l}$

(6) $$\eta_{l}=\frac{\left|A_{l}^{f}-A_{l}^{g}\right|}{A_{l}^{f}}$$

(iii) According to the improved Sigmoid function, i.e., Equation (7), the magnitude fault degree of the line *l* is obtained by calculating $\eta_{l}$, i.e., $A_{l}$.

(7) $$A_{l}=\frac{1}{1+e^{-6\eta_{l}}}-0.1$$

(c) Meteorological fault degree

According to Equation (8), information features are extracted from the acquired meteorological data to obtain the meteorological fault degree corresponding to the suspected faulty line, i.e., $R_{l}$.

(8) $$R_{l}=1-\prod_{i=1}^{h}\left(1-R_{i}\right)$$

where $R_{l}$ denotes the meteorological fault degree of line *l*; $R_{i}$ denotes the fault rate of a certain meteorological level of the i-th meteorological factor; *h* denotes the number of meteorological factors.

Step (3): Multi-source information fault degree fusion to obtain the integrated fault degree of the line.

(i) The weight value of each source is solved according to the analytic hierarchy process, i.e., $W_{i}$.

(ii) The integrated fault degree of the line is solved according to Equation (9), i.e., $G_{l}$.

(9) $$G_{l}=\sum_{i=1}^{q}W_{i}Q_{i}$$

where $G_{l}$ denotes the integrated fault degree of line *l*; $W_{i}$ denotes the weight value corresponding to the i-th source; $Q_{i}\left(1\leq i\leq q\right)$ denotes the fault degree of the i-th source; and *q* denotes the number of sources with a value of 3.

Step (4): Output diagnosis result. The status of the line is determined according to the integrated fault degree. If the integrated fault degree is greater than 0.5, then its corresponding transmission line has a fault; otherwise, it is not faulty.

## 4. Experimental Results and Analysis

**Figure 2:**
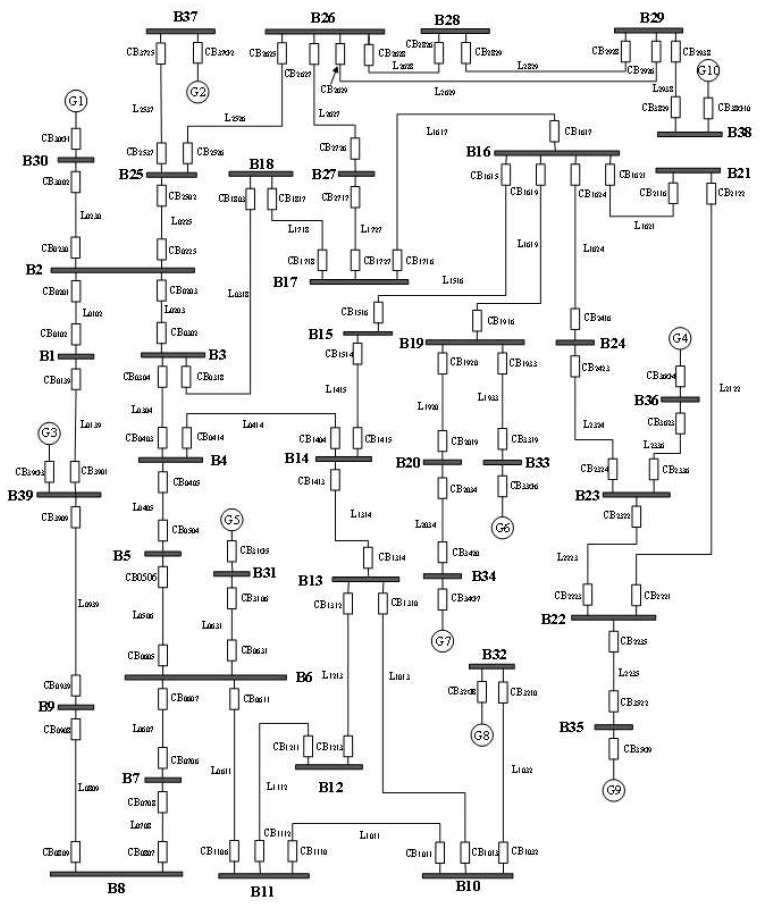
Standard IEEE39-bus system.

In this section, the power system simulation software PSCAD is used as a simulation modeling tool to build the standard IEEE39-bus system shown in Figure 2, which is used as a diagnostic object for verifying the feasibility and effectiveness of the method in this paper. The meteorological factors involved in this section and their corresponding failure rates for different meteorological levels are shown in Table 2. For both the case comparison study and the accuracy test, this section is based on the actual thunderstorm climate in a certain area as the external meteorological conditions of transmission lines, including lightning, rainfall, and wind, all of which have an orange meteorological rating.

### 4.1. Case Studies

Based on the above six case scenarios of thunderstorm climate, the diagnosis results of this paper’s method and the other two fault diagnosis methods are compared as shown in Table 3. From the table, we can see that for cases 1–3, all three methods can diagnose the faulty line accurately. For cases 4–5, both this paper and Ref. [26] can diagnose the correct fault line, while Ref. [27] fails to diagnose the fault line accurately because it cannot handle the wrong fault information well and produces multiple diagnostic results. For case 6, both the method in this paper and Ref. [27] can diagnose the correct fault line, while Ref. [26] fails to diagnose the fault line accurately due to the missing fault information. In addition, the diagnosis results of the method in this paper include the evaluation of fault information. Therefore, the method has good feasibility and effectiveness in transmission line fault diagnosis.

To illustrate the proposed method, case 1 is examined in detail.

Fault information of case 1: operated relays and tripped: main protection BR1, circuit breakers CB0102 and CB0139, and the voltage waveform data in telemetry is complete. Meteorological information of case 1: Lightning (orange), rainfall (orange), wind (orange).

First, according to the junction analysis method for case 1, it is determined that the suspected fault line includes bus $B_{1}$ and lines $L_{0102},\;\;L_{0225},\;\;L_{0203},L_{0230},\;\;L_{0139},\;\;L_{0939}$. Bus $B_{1}$ is used as an example for the introduction of the detailed calculation process.

Next, the fault degree of each source is calculated respectively, including:

(1) The fault degree of the switching quantity, i.e., the pulse fault degree.

First, a fault diagnosis model based on brSNPS is established for the suspected faulty line. Then according to the fault information, we obtain the initial pulse value vector of input neurons, i.e., $\theta_{p.0}=\left[1,1,1,0,0,0,0,0_{7}\right]$.

Then, the brSNPS matrix reasoning algorithm is performed to obtain pulse values of output neurons of the diagnosis model of bus $B_{1}$.

When g=0, we obtain the results

$$\theta_{r.0}=\left[0_{7}\right]$$

$$\theta_{p.0}=\left[1,\;1,\;1,\;0,\;0,\;0,\;0,\;0_{7}\right]$$

When g=1, we obtain the results

$$\theta_{r.1}=\left[1,\;1,\;0,\;0,\;0_{3}\right]$$

$$\theta_{p.1}=\left[0_{7},\;1,\;1,\;0,\;0,\;0_{3}\right]$$

When g=2, we obtain the results

$$\theta_{r.2}=\left[0_{4},\;1,\;1,\;0\right]$$

$$\theta_{p.2}=\left[0_{11},\;1,\;1,\;0\right]$$

When g=3, we obtain the results

$$\theta_{r.3}=\left[0_{6},\;1\right]$$

$$\theta_{p.3}=\left[0_{13},\;1\right]$$

When g=4, we obtain the results

$$\theta_{r.4}=\left[0_{7}\right]$$

Consequently, the termination condition is satisfied and the reasoning process ends. The brSNPS output neuron exports its pulse value, i.e., 1, denoting the pulse fault degree $\theta_{B_{1}}=1$ of the suspected fault bus $B_{1}$. (2) The degree of failure of an electrical quantity, i.e., the amplitude fault degree.

**Figure 3:**
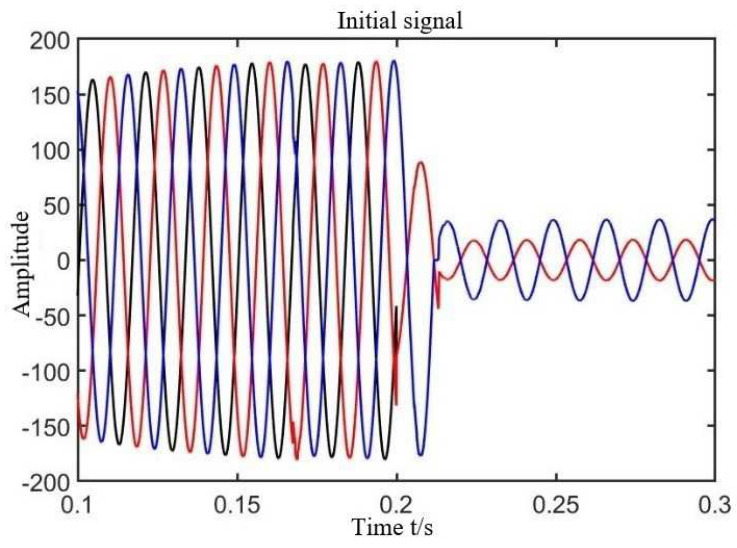
Telemeasurement waveform of $B_{1}$.

**Figure 4:**
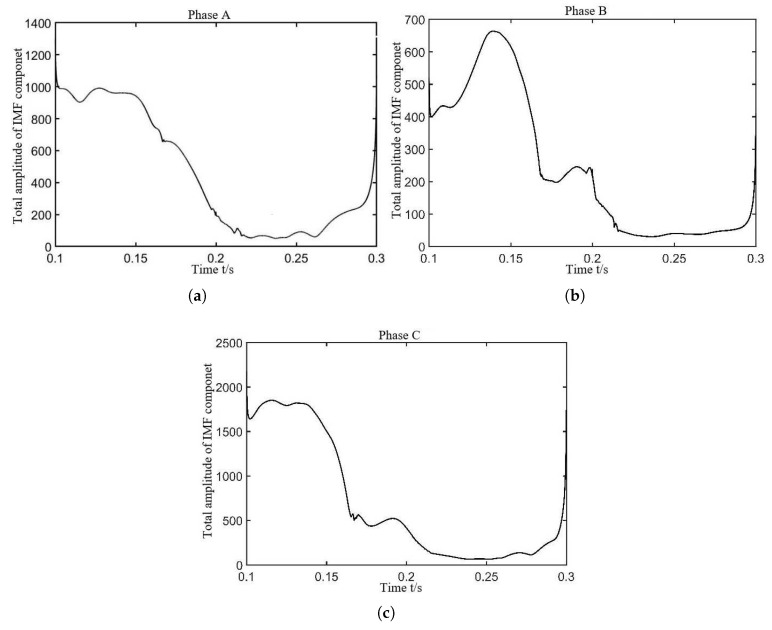
Total amplitude of IMF component of $B_{1}$. (**a**) Phase A. (**b**) Phase B. (**c**) Phase C.

The fault telemetry information of the suspected faulty bus $B_{1}$, i.e., the waveform of the voltage signal, is obtained according to the PSCAD simulation software simulation as shown in Figure 3, which is HHT and the total amplitude of each phase IMF component of bus $B_{1}$ is calculated according to Equations (4) and (5), as shown in Figure 4.

The amplitude fault degree of the suspected faulty bus $B_{1}$, i.e., $A_{B_{1}}=0.9$, is then calculated according to Equations (6) and (7).

(3) The degree of failure of meteorological data, i.e., the meteorological degree.

Based on the real-time meteorological conditions given in case 1 and the data in Table 2, the failure rates of bus $B_{1}$ under the three meteorological factors of lightning, rainfall and wind are obtained as 0.70, 0.21 and 0.44, respectively. The meteorological failure degree of the suspected faulty bus $B_{1}$, i.e., $R_{B_{1}}=0.8673$, is then calculated according to Equation (8). Then, the weight vectors of pulse fault degree, amplitude fault degree and meteorological fault degree of the suspected faulty bus $B_{1}$, i.e., W=[0.491,0.491,0.018], are calculated according to the hierarchical analysis method. Finally, the integrated fault degree of bus $B_{1}$, i.e., $G_{B_{1}}=0.9485$, is calculated according to Equation (9), denoting bus $B_{1}$ has a fault with an integrated fault degree confidence level of 0.9485. At the same time, the action information of the protection device is evaluated, and the protection device operates correctly and the uploaded information is error-free.

Similarly, the integrated fault degree $G_{L_{0102}}=0.0158,\;G_{L_{0225}}=0.0163,\;G_{L_{0203}}=0.0158,\;G_{L_{0230}}=0.0161,\;G_{L_{0319}}=0.0172,\;G_{L_{0939}}=0.0154$ of other suspected fault lines is calculated, denoting that line $L_{0102},\;L_{0225},\;L_{0203},\;L_{0230},L_{0139},\;L_{0939}$ are not faulted.

### 4.2. Accuracy Tests

To verify the diagnostic capability of the method in this paper, in the case of distortion and loss of fault information, the fault information uncertainty ratio is defined, i.e., Equation (10).

(10) $$\gamma =\frac{\upsilon^{r}}{\upsilon^{f}}\times 100$$

where γ denotes the line fault information uncertainty ratio; $\upsilon^{r}$ is the number of line uncertainty fault information; $\upsilon^{f}$ is the total number of line fault information, including switching information and electrical information. It is noteworthy that the information uncertainty ratio is a random mixture of a certain amount of uncertain information in the correct fault information, where the uncertain information is obtained by simulating the rejection and misoperation of the protection device, random changes in the sampling point value of the recording equipment, and the distortion and loss of information during signal transmission.

The accuracy test in this section takes bus B1 in the standard IEEE39-bus system as the test object, and every time we change the proportion of uncertain information in the fault information, i.e., γ value, we rediagnose the fault information to get the diagnostic accuracy of the methods with different γ values. The test results of the three fault diagnosis methods from γ=0 to γ=10 are shown in Table 4, where each diagnostic accuracy is obtained by 10,000 tests. As can be seen from Table 4, the accuracy of all three methods is 100% when γ=0. (i.e., all fault information is correct). However, due to meteorological conditions, the accuracy of the proposed method remains high as γ increases. Note: For the value of γ in Table 4, the fault information data used in the proposed method takes the same value for both switching and electrical quantities, while Refs. [26,27] take this value for the corresponding electrical quantity information or switching quantity information individually.

## 5. Conclusions

To improve the accuracy and fault tolerance of transmission lines, this paper proposes a fault diagnosis method for a transmission network based on multi-source information fusion. The integrated fault diagnosis based on switching, electrical and meteorological information effectively solves the problem of low fault tolerance of fault diagnosis using only switching quantities. The diagnosis method models for a single line and reduces the complexity of the model. The electrical quantity fault degree criterion is established by the improved Sigmoid function, which reduces the use of electrical quantity information and improves the efficiency of the diagnosis method. By introducing meteorological information, the negative impact of meteorological environment on fault information is further eliminated. The comprehensive use of multi-source information improves the diagnostic accuracy and fault tolerance of the method. The fault cases as well as the results of the accuracy comparison demonstrate its feasibility and effectiveness.

Although the results obtained on the IEEE 39-bus benchmark system demonstrate the effectiveness of the proposed method, further validation using physical platforms and real-world power system data is needed. Future work will focus on evaluating the proposed method in more realistic operating environments to further verify its practical applicability. In addition, comparisons with advanced data-driven fault diagnosis methods, including CNN- and GNN-based approaches, will be investigated using larger-scale fault datasets to further assess the advantages and limitations of the proposed method. 

## Figures and Tables

**Table 1:** Table of the relationship between every two factors.

Number	Important Level
1	Equally important
3	Slightly important
5	Obviously important
7	Strongly important
9	Extremely important
2, 4, 6, 8	Between above level

**Table 2:** Fault rates of three meteorological factors under different levels.

Meteorological	Meteorological Levels				
**Factors**	**Red**	**Orange**	**Yellow**	**Blue**	**Level 1**
Thunder					
and	0.84	0.72	0.52	0.16	0.0038
Lightning					
Rainfall	0.42	0.21	0.08	0.05	0.0039
Wind	0.64	0.44	0.24	0.12	0.0014

**Table 3:** Comparisons of diagnosis results between the proposed method and two fault diagnosis methods.

**Cases**	Fault Information		Actual	Ref. [26]	Ref. [27]	Fault Information	
	Protective Relays	Circuit Breakers	Fault Lines			Diagnosis Results	Information Evaluation
1	${\mathrm{BR}}_{1}$	${\mathrm{CB}}_{0102}$,${\mathrm{CB}}_{0139}$	$B_{1}$	$B_{1}$	$B_{1}$	$B_{1}$	Correct Action
2	${\mathrm{BR}}_{1},{\mathrm{MLR}}_{1817}$	${\mathrm{CB}}_{0102},{\mathrm{CB}}_{0139}$	$B_{1}$	$B_{1}$	$B_{1}$	$B_{1}$	Correct Action
	${\mathrm{MLR}}_{1718}$	${\mathrm{CB}}_{1718}$,${\mathrm{CB}}_{1817}$	$L_{1718}$	$L_{1718}$	$L_{1718}$	$L_{1718}$	
3	${\mathrm{BR}}_{1},{\mathrm{SLR}}_{0201}$	${\mathrm{CB}}_{0139},{\mathrm{CB}}_{0201}$	$B_{1}$	$B_{1}$	$B_{1}$	$B_{1}$	Error
	${\mathrm{MLR}}_{1817},{\mathrm{MLR}}_{1718}$	${\mathrm{CB}}_{1718},{\mathrm{CB}}_{1817}$	$L_{1718}$	$L_{1718}$	$L_{1718}$	$L_{1718}$	Message: ${\mathrm{SLR}}_{1803}$
	${\mathrm{SLR}}_{1803}$						
4	${\mathrm{BR}}_{1},{\mathrm{SLR}}_{0201}$	${\mathrm{CB}}_{0139},{\mathrm{CB}}_{0201}$	$B_{1}$	$B_{1}$	$B_{1}$	$B_{1}$	Error Message: ${\mathrm{BR}}_{18}$,
	${\mathrm{BR}}_{18}$	${\mathrm{CB}}_{1803},{\mathrm{CB}}_{1817}$			$B_{18}$		${\mathrm{CB}}_{1803},{\mathrm{CB}}_{1817}$
5	${\mathrm{BR}}_{1}$	${\mathrm{CB}}_{0102},{\mathrm{CB}}_{0139}$	$B_{1}$	$B_{1}$		$B_{1}$	Miss Message:
	${\mathrm{MLR}}_{1817}$	${\mathrm{CB}}_{1817}$,${\mathrm{CB}}_{0318}$	$L_{1718}$	$L_{1718}$	$B_{1}$	$L_{1718}$	${\mathrm{MLR}}_{1718}$,${\mathrm{CB}}_{1718}$
							Error Message: ${\mathrm{CB}}_{0318}$
6	${\mathrm{BR}}_{1}$	${\mathrm{CB}}_{0102}$,${\mathrm{CB}}_{0139}$	$B_{1}$	$B_{1}$	$B_{1}$	$B_{1}$	Miss Message:
	${\mathrm{MLR}}_{1817}$,${\mathrm{MLR}}_{1718}$	${\mathrm{CB}}_{1817}$,${\mathrm{CB}}_{1718}$	$L_{1718}$		$L_{1718}$	$L_{1718}$	Voltage Waveform of $L_{1718}$

**Table 4:** Accuracy of fault diagnosis at different values of γ.

γ	The Proposed Method	Ref. [26]	Ref. [27]
0%	100%	100%	100%
1%	99.90%	99.68%	99.16%
2%	99.84%	99.59%	98.35%
3%	99.77%	99.44%	97.13%
4%	99.72%	99.39%	96.94%
5%	99.69%	99.33%	96.12%
6%	99.63%	99.25%	95.35%
7%	99.52%	99.13%	94.57%
8%	99.35%	98.83%	93.91%
9%	99.25%	98.67%	93.18%
10%	99.16%	98.46%	92.40%

## Data Availability

Data will be made available on request.
